# WARP Interacts with Collagen VI-Containing Microfibrils in the Pericellular Matrix of Human Chondrocytes

**DOI:** 10.1371/journal.pone.0052793

**Published:** 2012-12-26

**Authors:** Uwe Hansen, Justin M. Allen, Rachel White, Cathleen Moscibrocki, Peter Bruckner, John F. Bateman, Jamie Fitzgerald

**Affiliations:** 1 Institute for Physiological Chemistry and Pathobiochemistry, University Hospital of Muenster, Muenster, Germany; 2 Murdoch Childrens Research Institute, Parkville, Victoria, Australia; 3 Department of Paediatrics, and University of Melbourne, Parkville, Victoria, Australia; 4 Biochemistry and Molecular Biology, University of Melbourne, Parkville, Victoria, Australia; 5 Department of Orthopaedics and Rehabilitation, Oregon Health and Science University, Portland, Oregon, United States of America; University of California, San Diego, United States of America

## Abstract

Collagen VI and WARP are extracellular structural macromolecules present in cartilage and associated with BM suprastructures in non-skeletal tissues. We have previously shown that in WARP-deficient mice, collagen VI is specifically reduced in regions of the peripheral nerve ECM where WARP is expressed, suggesting that both macromolecules are part of the same suprastructure. The object of this study was to conduct a detailed analysis of WARP-collagen VI interactions *in vitro* in cartilage, a tissue rich in WARP and collagen VI. Immunohistochemical analysis of mouse and human articular cartilage showed that WARP and collagen VI co-localize in the pericellular matrix of superficial zone articular chondrocytes. EM analysis on extracts of human articular cartilage showed that WARP associates closely with collagen VI-containing suprastructures. Additional evidence of an interaction is provided by immunogold EM and immunoblot analysis showing that WARP was present in collagen VI-containing networks isolated from cartilage. Further characterization were done by solid phase binding studies and reconstitution experiments using purified recombinant WARP and isolated collagen VI. Collagen VI binds to WARP with an apparent K_d_ of approximately 22 nM and the binding site(s) for WARP resides within the triple helical domain since WARP binds to both intact collagen VI tetramers and pepsinized collagen VI. Together, these data confirm and extend our previous findings by demonstrating that WARP and collagen VI form high affinity associations *in vivo* in cartilage. We conclude that WARP is ideally placed to function as an adapter protein in the cartilage pericellular matrix.

## Introduction

The extracellular matrix (ECM) is composed of networks with unique functional and biological characteristics that are formed by specific macromolecular suprastructures composed of proteins, glycoproteins, proteoglycans, and glycosaminoglycans. A detailed understanding of how these components interact is important for elucidating the pathobiology of diseases that involve the ECM. Defining the major protein-protein interactions in connective tissues provides important insights into specific developmental processes and for interpreting transgenic and knock-out mouse phenotypes. The goal of this study is to characterize the molecular interaction between von Willebrand factor A-domain related protein (WARP) [1], [2], [3], [4] and the ubiquitous ECM macromolecule, collagen VI. The rationale for this study came from our finding that in mice null for *Vwa-1*, the gene for WARP, collagen VI is reduced suggesting a direct functional relationship between the two ECM components VI [3].

Six genetically distinct collagen VI chains, α1(VI), α2(VI), α3(VI), α4(VI), α5(VI) and α6(VI), encoded by the *COL6A1* to *COL6A6* genes, are now known to exist [5], [6], [7], [8], [9]. Like all collagens, these chains initially assemble into trimeric structures. Heterotrimers of the α1(VI), α2(VI), α3(VI) chains are known to assemble into microfibrillar structures by a unique hierarchical process [10], [11]. The molecular and suprastructural associations of the recently described α4(VI), α5(VI) and α6(VI) chains are not yet established. Collagen VI is integrated in many tissues into abundant and structurally unique microfibrils in close association with basement membranes. Several recent studies suggest that such microfibrils tether basement membranes to the interstitial matrix [12], [13]. This hypothesis is supported by the findings that collagen VI interacts specifically with several macromolecules of basement membranes or the interstitial extracellular matrix, including perlecan [14], collagen IV [15], βig-h3 [16], and NG2 [17] or fibrillar collagens [18], biglycan, and decorin [19], respectively.

In cartilage, collagen VI is an abundant component of the chondrocyte pericellular matrix (PCM) [20], a basement membrane-like structure [21]. Atomic force microscopy experiments demonstrated that collagen VI is a major contributor to the biomechanical integrity of the PCM [22]. A biomechanical role for collagen VI in articular cartilage is further supported by the finding that mice null for the *Col6a1* gene demonstrate reduced biomechanical characteristics [23].

Human WARP is a 50 kDa protein encoded by the *VWA1* gene [4]. Biochemical studies demonstrate that WARP oligomerizes to form large disulfide-bonded multimeric structures in cartilage. During development, WARP is expressed within presumptive articular cartilage prior to joint cavitation and is present in the PCM of developing elements of articular and fibrocartilage including intervertebral disc, sternal cartilage and meniscus [2]. Further studies using a mouse line expressing a reporter gene at the *Vwa1* locus demonstrated that, in addition to cartilage, WARP is expressed near basement membrane structures in several tissues including the peripheral nervous system, the apical ectodermal ridge of developing limb buds, and skeletal and cardiac muscle [1]. Consistent with a basement membrane role for WARP is the finding that it forms high affinity associations with perlecan, a proteoglycan prominently occurring in the cartilage pericellular matrix [24] and in basement membrane [2].

We previously reported that the primary phenotype of the WARP-null mouse is a peripheral nerve abnormality that manifests as a delayed response to acute painful stimulus and impaired fine motor coordination [3]. The main biochemical phenotype is a reduction and mislocalization of collagen VI in the endoneurium where WARP is expressed, but not in the outer perineurium layer where WARP is not expressed, suggesting that the reduction in collagen VI is directly related to the absence of WARP protein. Support for the hypothesis that WARP and collagen VI associated directly was provided by a surface plasmon resonance experiment [3]. Here, we expand the analysis of the WARP-collagen VI interaction with *in vivo* and *in vitro* experiments including immunohistochemistry, solid phase binding studies, electron microscopy analyses and a novel approach for isolation and analysis of matrix suprastructures. The *in vivo* experiments are focused on normal human cartilage, a tissue where both WARP and collagen VI are known to be expressed in close association with the PCM.

## Materials and Methods

### Immunohistochemistry

Paraffin embedded sagittal femoral cartilage sections from healthy individuals were treated with hyaluronidase (2 mg/ml; from bovine testes; Sigma Aldrich) and incubated with 0.2% glycine for 1 h. The slides were then blocked with 0.1% horse serum and 1% (w/v) bovine serum albumin (Fraction V; Fisher) and incubated overnight at 4°C with rabbit anti-human collagen VI (pAb, Fitzgerald Industries) diluted 1∶200 and sheep anti-WARP (pAb, whole molecule) diluted 1∶500 in Antibody Diluent (Dako). After washing bound collagen VI antibodies were detected with goat anti-rabbit Texas Red (BD Bioscience) diluted 1∶500 and bound WARP antibodies were detected with donkey anti-sheep AlexaFluor647 (BD Biosciences). The sections were mounted in 40,6-diamidino-2-phenylindole (DAPI)-infused anti-fade mounting media (Prolong Gold with DAPI; Invitrogen) and visualized with a high resolution wide field microscope, based on the Olympus IX71 microscope with DIC transmitted light, mounted with a Nikon Coolpix HQ camera. The far-red fluorophore Alexafluor647 used to detect WARP was switched to green for co-localization analysis with imaging processing software (softWoRx Explorer).

Knee joints from 8-week-old mice were fixed with 4% paraformaldehyde in PBS, and decalcified in PBS containing 7% (w/v) EDTA. Tissues were frozen in Tissue Tek OCT compound and 10 µm sections of tibial articular cartilage were prepared. Sections were blocked and permeabilized with PBS containing 1% BSA and 0.1% Triton-X 100 for 1 h, then immunolabeled overnight at 4°C with a sheep polyclonal antibody raised against full length recombinant WARP [1] and rabbit anti-collagen VI (Fitzgerald Industries). Controls were probed with pre-immune serum or with secondary antibodies only. Bound antibodies were detected with donkey anti-sheep Alexa Fluor 488 and donkey anti-rabbit Alexa Fluor 594 secondary antibodies (Invitrogen). The slides were counterstained with DAPI (4′,6-diamidino-2-phenylindole), washed with PBS and mounted in Fluorsave (Calbiochem). Immunofluorescence images were visualized by laser scanning confocal microscopy using a Leica TCS SP2 SE confocal microscope.

### Extraction of authentic suprastructures and immunoelectron microscopy

Fragments of suprastructural aggregates were obtained from human articular cartilage recovered after joint replacement surgery by mechanical disruption of the tissue as described previously [25]. Aliquots of supramolecular fragments were absorbed to Formvar/carbon coated nickel grids, washed with PBS, and treated for 30 min with 2% (w/v) dried skim milk in PBS. Next, adsorbed material was allowed to react for 2 h with a sheep antibody against WARP (raised against full-length recombinant WARP) or chicken antibodies against WARP (raised against WARP C-terminal domain) and rabbit antibody against collagen VI (AB 7821, Millipore) or mouse antibodies against collagen VI (AF 6210, MEDICORP Inc.) diluted 1∶100 in PBS containing 0.2% dry milk. After washing with PBS, grids were incubated with 0.2% (w/v) milk solution containing colloidal gold particles coated with anti-rabbit or anti-mouse immunoglobulins (12-nm gold particles) and ant-sheep or anti-chicken immunoglobulins (18-nm gold particles) (Jackson Immuno Research) diluted 1∶30. Finally, grids were washed with distilled water and negatively stained with 2% uranyl acetate. Control experiments were done with first antibodies omitted, and in addition, with pre-immune serum controls. Electron micrographs were taken at 60 kV with a Philips EM 410 electron microscope.

### Ultrathin sections of articular cartilage

Freshly obtained articular cartilage of adult mice was fixed in 4% paraformaldehyde and 0.25% glutaraldehyde in 100 mM sodium cacodylate buffer, pH 7.4, at 4°C and decalcified in Tris-buffer containing 10% (w/v) EDTA (pH 7.4). Samples were rinsed in PBS, dehydrated in ethanol up to 70%, and embedded in LR White embedding medium (London Resin Company, UK). Ultrathin sections were cut and collected on Formvar/carbon coated nickel grids for immuno-gold EM. The sections were incubated with 100 mM glycine in PBS for 2 min, washed twice with PBS and incubated with 2% dried skim milk in PBS for 30 min. Polyclonal antibodies against WARP and monoclonal antibodies against collagen VI, both diluted 1∶50 in 0.2% dried skim milk, were incubated for 2 h. After washing with PBS, sections were incubated with gold-conjugated antibodies against sheep- (18-nm gold particles) and mouse-immunoglobulins (12-nm gold particles) diluted 1∶30 in 0.2% (w/v) dried skim milk. Finally, sections were rinsed with water and stained with uranyl acetate. As a control primary antibodies were omitted.

### Reconstitution experiments

Recombinant WARP was purified from the medium of EBNA-293 cells [2]. Small aliquots of recombinant WARP were biotinylated with EasyLink Biotin (type A) conjugation kit (Abcam). For binding experiments, aliquots of isolated collagen microfibrils were incubated with recombinant WARP (dimer) or biotinylated recombinant WARP (dimer or multimer) for 1 h. Aliquots were absorbed to nickel grids coated with glow-discharged carbon film, and washed with 20 mM Tris-HCl, containing 0.15 M NaCl, pH 7.4 (TBS) and negatively stained with uranyl formiate. In some experiments, biotinylated WARP was visualized by gold labeled streptavidin (5-nm gold particles; KPL, USA) and negatively stained with uranyl formiate and analyzed by EM.

### Solid-phase binding assays

Polystyrene microtiter plates were coated overnight at 4°C with 10 µg of purified collagen VI tetramers in 100 µl TBS. After washing with TBS, wells were blocked with 2% (w/v) bovine serum albumin in TBS at room temperature prior to further washing with TBS containing 0.04% Tween 20 (TBS-T). Recombinant WARP was then added at a concentration ranging between 0–1 µM dissolved in 100 µl of TBS. After incubating for 1 h at room temperature the wells were washed with TBS-T. Bound WARP was subsequently detected with anti-sheep serum against WARP followed by a horseradish peroxidase-conjugated antibody raised against sheep immunoglobulins. Absorbance at 405 nm was measured using a microplate reader.

For the reciprocal binding experiment, plates were coated overnight at 4°C with 5 µg/ml of recombinant WARP (fractions containing dimer, mix of dimer and multimer, and primarily multimer as shown in Fig. S1) in 100 µl TBS. After blocking, purified collagen VI tetramers were added from 1 µg/ml to 30 µg/ml dissolved in 100 µl of TBS, and washed with TBS-T. Bound collagen VI tetramers were detected with a rabbit antibody against collagen VI (Ab 7821, Millipore) followed by horseradish peroxidase-conjugated secondary antibodies against rabbit immunoglobulins. Absorbance was measured at 490 nm. In addition, binding experiments with recombinant WARP dimer were performed using pepsin-digested collagen VI tetramers and microfibrils. Aliquots were reduced with DTT prior digestion with pepsin overnight at 4°C. After centrifugation, the obtained supernatant was further purified on a Superose 6 column.

### Isolation of collagen VI microfibrils

Native collagen VI microfibrils or tetramers were isolated from bovine cornea [26]. In brief, corneas were cut into small pieces and homogenized with a Polytron in 50 mM TrisHCl, 400 mM NaCl, 10 mM CaCl_2_, pH 7.4, and protease inhibitors. The resulting homogenate was digested (20 mg/g tissue, bacterial collagenase type I, Worthington) overnight at RT. After centrifugation, supernatant was filtrated and aliquots were fractionated on a Superose 6 column. In addition, aliquots of isolated collagen VI microfibrils were dialyzed against 100 mM sodium citrate, pH 4.0, at 4°C to de-polymerize collagen VI microfibrils into tetramers. The resulting tetramers were again dialyzed against Tris-buffer and fractionated. Purity of the isolated material was confirmed by SDS-PAGE and immunoblotting (see Fig. S1).

### Pull-down experiments using superparamagnetic immunobeads

Extracts from articular cartilage containing suprastructural fragment were used for the isolation by superparamagnetic immunobeads (Invitrogen) [20]. Superparamagnetic polysterene beads covered with affinity-purified secondary antibodies (e.g. sheep anti-mouse immunoglobulins) were covalently coupled to primary antibodies against collagen VI (AF 6210). As control for specificity of the separation procedure immunobeads were coated with normal mouse serum (Dako). The bead pellets of each separation step were washed several times with PBS and re-suspended in SDS-PAGE loading buffer containing 5% (v/v) β-mercaptoethanol. The re-suspended pellets were pooled and analyzed by immunoblotting.

### Immunoblotting

SDS-PAGE was carried out using 4.5–15% polyacrylamide gradient gels under reducing conditions. For immunoblotting, proteins were electro-transferred to nitrocellulose filters and incubated with antibodies against WARP (sheep ab raised against recombinant WARP or chicken ab against WARP C-terminal domain, both diluted 1∶1000) or collagen VI (Ab 7821, Millipore, diluted 1∶5000), followed by HRP-coupled anti-rabbit, -sheep or -chicken antibodies (DAKO). For total protein detection, nitrocellulose membranes were stained with MemCode™ reversible stain kit (ThermoScientific). Immunoreactivity was revealed by chemiluminescence (ECL, Pierce).

## Results and Discussion

**Figure 1:**
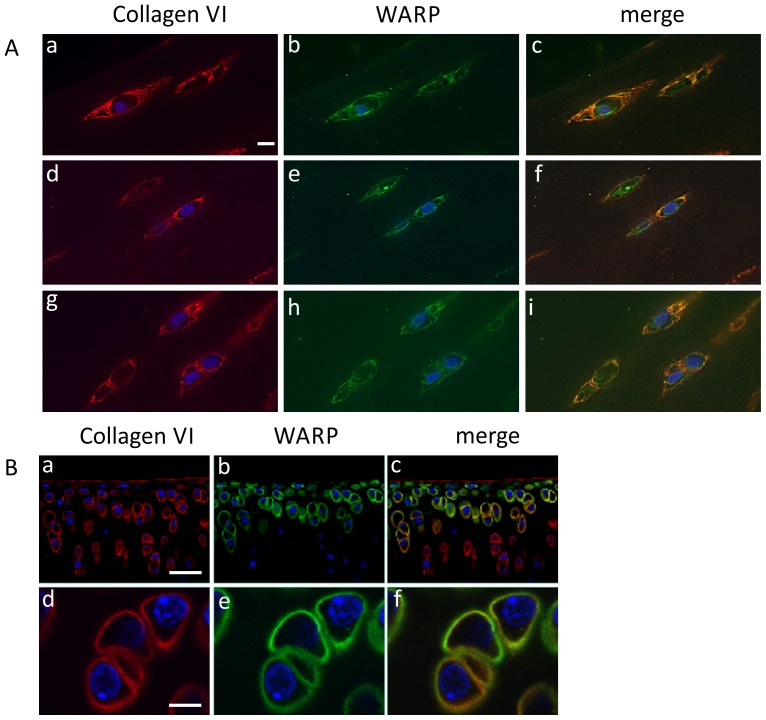
Immunohistochemical localization of WARP and collagen VI in articular cartilage. A, Superficial zone human articular cartilage was stained for collagen VI (panels a, d and g) and WARP (panels b, e and h). The merged images show clear co-localization of collagen VI and WARP in the pericellular environment (panels c, f and i). The scale bar shown in panel (a) is 100 µm. B, Mouse tibial articular cartilage stained with collagen VI (panels a and d) and WARP antisera (panels b and e). Merged images show co-localization of collagen VI and WARP in the chondrocyte pericellular matrix (panels c and f). Scale bar in panel (a) is 200 µm and in (d) 50 µm.

**Figure 2:**
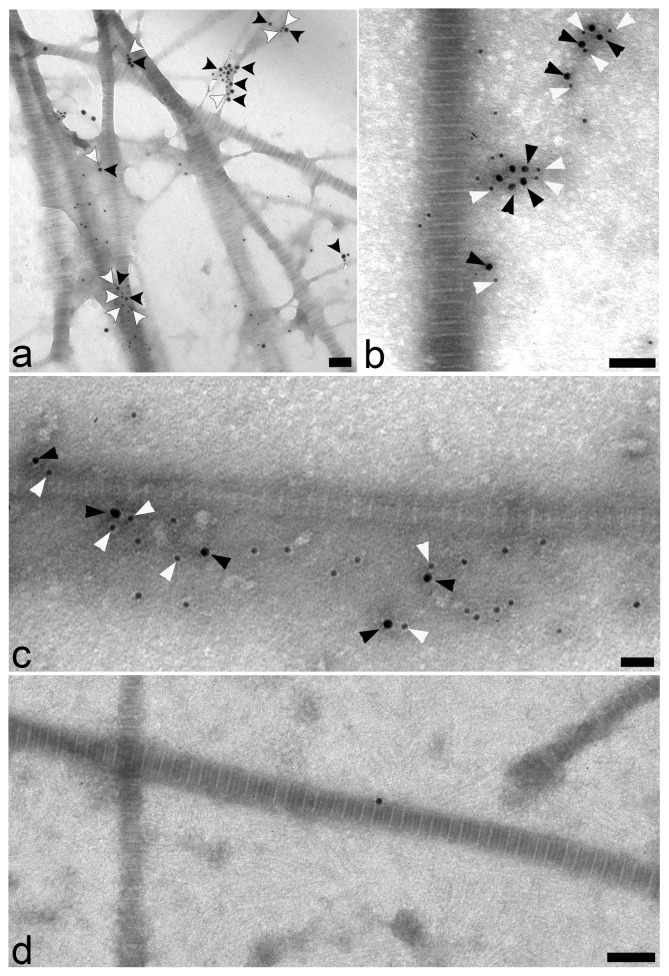
Localization of WARP and collagen VI using electron microscopy. EM analysis on native supramolecular fragments isolated from human articular cartilage. Sheep antisera against WARP (18-nm gold particles; black arrow heads) (panels a–c) and a rabbit polyclonal anti-collagen VI (panels a and b) or monoclonal antibody against collagen VI (panel c) (12-nm gold particles; white arrowheads) was used. A secondary antibodies only control is shown in panel d. Scale bars: 100 nm.

**Figure 3:**
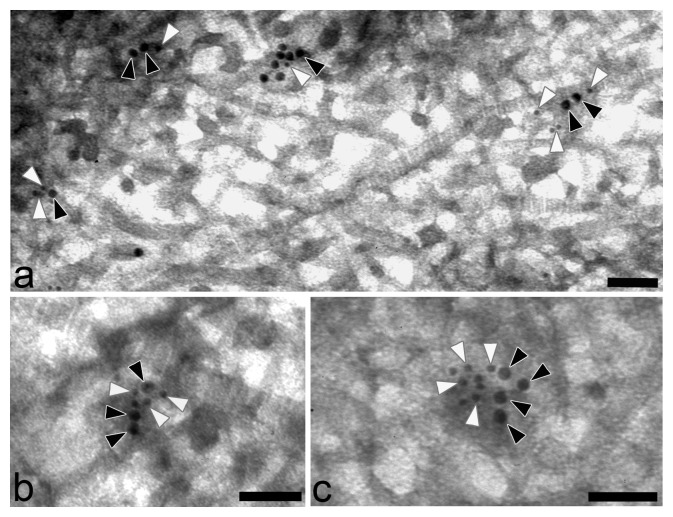
Representative ultrathin sections of articular cartilage of adult mice co-stained for WARP (18-nm gold particles; black arrow heads) and collagen VI (12-nm gold particles; white arrowheads). Scale bars: 100 nm.

To assess whether WARP and collagen VI co-localize in cartilage, we conducted immunohistochemistry and confocal microscopy on human and mouse articular cartilage (Fig. 1) and immune-gold EM studies on cartilage extracts (Fig. 2) and ultrathin cartilage sections (Fig. 3). Antibodies against collagen VI and WARP demonstrate that both macromolecules are present in the pericellular matrix (PCM) surrounding superficial zone chondrocytes in human articular cartilage. The merged images clearly show co-localization of the two macromolecules throughout most of the PCM (chondron) (Fig. 1A). In mouse, WARP expression is restricted to the superficial and intermediate zones of articular cartilage (Fig. 1B). Collagen VI is mostly absent from superficial layers but is present in the middle and deep articular cartilage zones (upper panels). In the region of overlap in the intermediate layer WARP and collagen VI co-localize in the pericellular matrix (lower panels). The reason for the differences in pericellular location of collagen VI and WARP between humans and mice is not known but may be related to differential biomechanical requirements within the joints of the two species.

Further evidence that WARP and collagen VI exist in close proximity in cartilage comes from immuno-gold EM experiments conducted on extracts of native suprastructural fragments isolated from human articular cartilage (Fig. 2). The extrafibrillar material was labeled with antibodies against WARP and collagen VI antibodies (panels a–c), confirming that they are closely associated in cartilage. The experiment was repeated using an antibody against the C-terminal domains of WARP and gave similar results (data not shown). A control experiment where primary antibodies were omitted is shown in panel d. When WARP primary antibodies were pre-absorbed with purified WARP protein no labeling was detected (data not shown). In addition, immuno-gold EM was performed on ultrathin sections of articular cartilage of adult mice (Fig. 3). Similar to the results using cartilage extracts, immuno-labeling of suprastructural aggregates for WARP and collagen VI occurred in close vicinity, confirming the *in-vivo* association of collagen VI and WARP.

**Figure 4:**
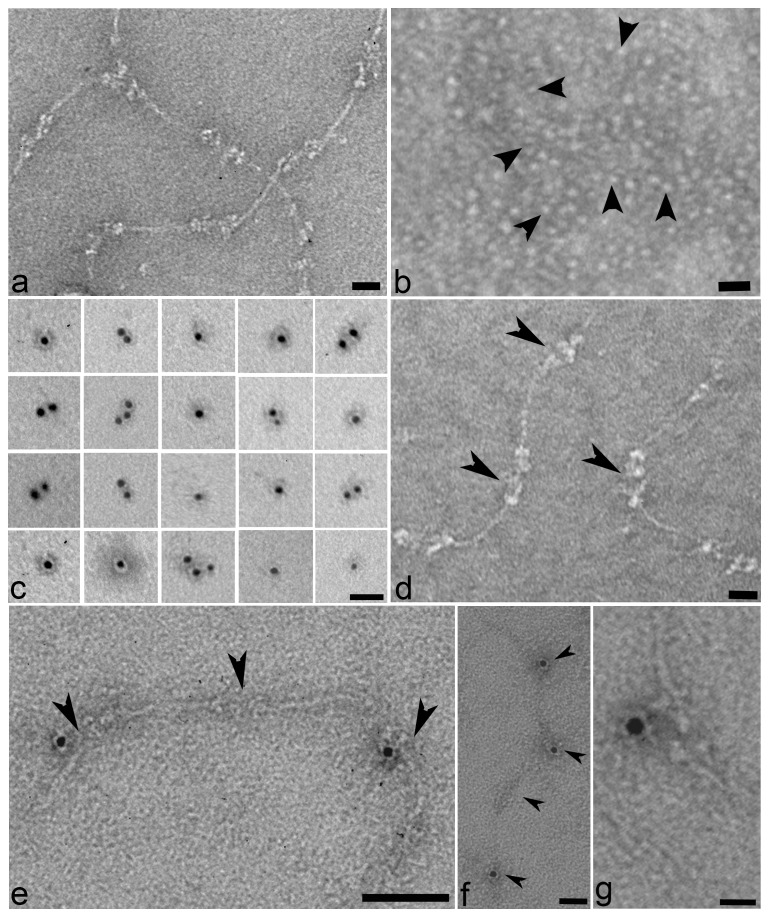
WARP binds to collagen VI *in vitro*. Isolated collagen VI microfibrils (shown in a) were mixed with recombinant WARP. Globular structures representing WARP are marked with arrowheads and visualized by negative staining (shown in b). Biotinylated recombinant WARP was visualized by gold labeled streptavidin as shown in panel c (5-nm gold particles). WARP is present on the collagen VI microfibrils (panel d) visible as structures close to the globular domains of the collagen VI microfibrils (bound WARP; black arrowheads). These structures are absent in the control experiments where WARP is omitted (see a). Biotinylated recombinant WARP (5-nm gold particles) bound near the junction between helical and globular domains of collagen VI microfibrils (panels e–g). Scale bars: 100 nm in a, b and d, 50 nm in e, 25 nm in c and f, and 10 nm in g.

The immunohistochemical and EM experiments presented so present evidence that WARP and collagen VI co-localize at the tissue level in articular cartilage. However, to assess whether WARP and collagen VI interact directly, reconstitution experiments were conducted using collagen VI microfibrils prepared from bovine cornea and recombinant human WARP (Fig. 4). SDS PAGE of collagen VI and WARP preparations is shown in Figure S1. Collagen VI microfibrils and WARP dimers were mixed and the reaction products were analyzed by negative staining and immuno-gold EM. A representative micrograph showing collagen VI microfibrils in the absence of recombinant WARP is shown in Figure 4A. Characteristic beaded microfibrils which contained short triple helical domains separated by globular domains were apparent. Unlike collagen VI isolated from Swarm rat chondrosarcoma cells under similar conditions [27], these microfibrils appear to lack bound ligands such as COMP, WARP, or matrilins which was also confirmed by immunoblotting (data not shown). The recombinant WARP preparation used in the reconstitution experiment is shown in Figure 4B. The presence of WARP in a negatively stained preparation was confirmed using biotinylated dimers or multimers of WARP detected with 5-nm-streptavidin-gold (Fig. 4C). When WARP dimers and collagen VI microfibrils are mixed the globular domains of the collagen VI microfibrils became conspicuously larger (arrowheads in Fig. 4D) suggesting that WARP is binding close to the junction between the triple helical and globular domains of collagen VI. Streptavidin-gold-EM analysis conducted on the WARP-collagen VI reconstitution mixture confirmed that WARP dimers bound to collagen VI in a regular spacing pattern that corresponded to the globular domains (Fig. 4E–G). WARP multimers did not bind to isolated collagen VI microfibrils (data not shown).

**Figure 5:**
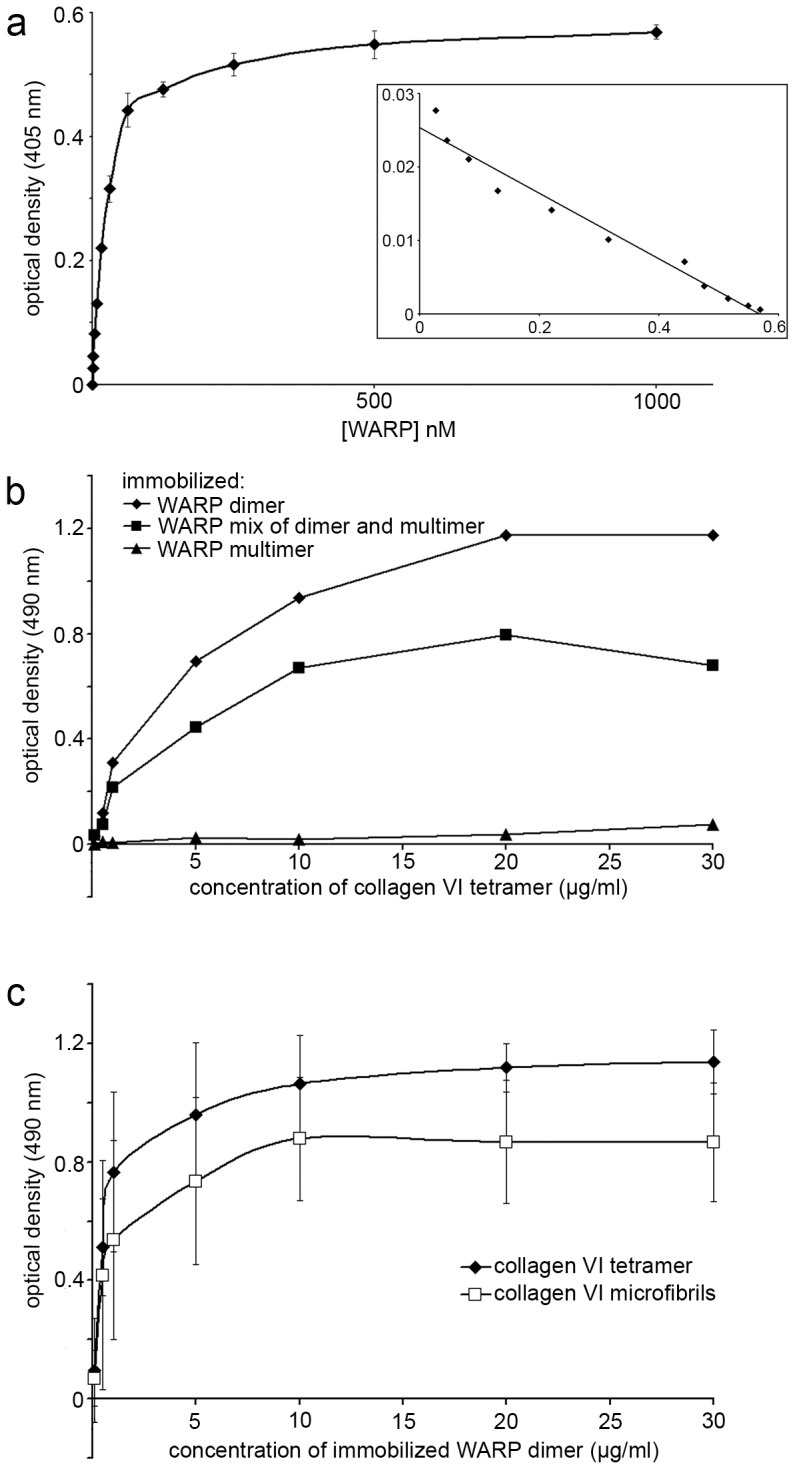
Solid phase analysis of WARP/collagen VI interaction. a, WARP binding to collagen VI tetramers. Coated collagen VI was incubated with recombinant WARP. Data are means of triplicate determinations +/− SE. *Inset*, Scatchard analysis of binding data reveals an apparent kDa of 22 nM. b, Reciprocal solid phase binding experiment showing collagen VI binding to coated WARP dimer but not WARP multimers. Representative curves are shown. c, WARP binds to both pepsinized collagen VI tetramers (black diamonds) and pepsinized intact collagen VI (white squares).

To demonstrate their direct interaction biochemically, collagen VI tetramers were coated onto microtiter plates and incubated with recombinant WARP dimer in solution. WARP exhibited saturable binding to the immobilized collagen VI (Fig. 5A) with an apparent K_d_ of 22 nM (*inset*) although the curve demonstrated a sigmoidal shape suggesting the possibility of more than one, cooperatively interacting binding site. The reciprocal binding experiment with immobilized WARP and soluble collagen VI tetramers was also conducted (Fig. 5B). Three different preparations of WARP were assessed for binding to collagen VI; WARP dimers, WARP multimers and a mix of WARP dimers and multimers (see Fig. S1, panel b for WARP fractions). Immobilized WARP dimers, but not multimers bound to collagen VI tetramers. A mix of dimers and multimers gave intermediate results indicating that WARP dimers are competent to associate with collagen VI tetramers *in vitro*, whereas WARP multimers are not. This finding is consistent with the WARP and collagen VI reconstitution experiments which demonstrated interaction of WARP dimers but not WARP multimer preparations with isolated collagen VI (see Fig. 4). The reason for this difference is not known. However, collagen VI tetramers may only be able to co-assemble with WARP before it undergoes multimerization. Also, the binding of WARP-multimers may be sterically unfavorable. An alternative explanation is that, depending on the nature of their binding partners, distinct WARP–oligomers may be amalgamated into discrete suprastructures, each exerting specific tissue functions.

To determine whether the binding site(s) for WARP resides in the collagen VI triple helix, pepsinized collagen VI tetramer and microfibrils were bound to different amounts of immobilized WARP dimer (Fig. 5C). Both pepsinized tetramer and microfibrils bound to WARP indicating that, consistent with the EM data (Fig. 4D–G), the WARP binding site(s) are located at the ends of the triple helical rods and/or in close proximity within the globular domains of collagen VI.

**Figure 6:**
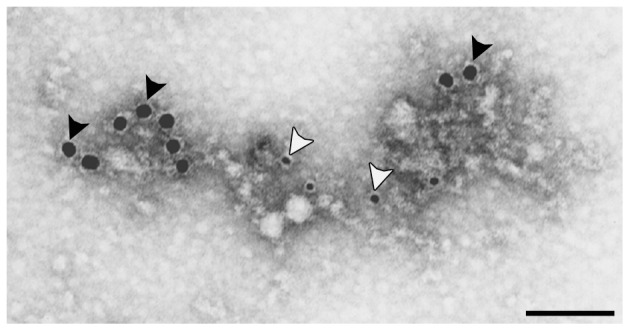
Collagen VI suprastructure analysis. A, Collagen VI suprastructures were isolated from human articular cartilage extracts using superparamagnetic immunobeads coupled to collagen VI antibodies. The isolated suprastructures were then doubly immuno-labeled with sheep anti-WARP antiserum (18-nm gold particles; black arrowheads) and rabbit anti-collagen VI antibody (small gold particles; white arrowheads). WARP is present in collagen VI suprastructures. Scale bar is 100 nm.

To assess whether WARP and collagen VI associate *in vivo*, we applied a technique developed recently to study interactions of matrix macromolecules at the suprastructural level [28]. Native collagen VI-containing suprastructures were isolated from human articular cartilage using magnetic immunobeads coated with antibodies to collagen VI. Large gold particles corresponding to WARP decorated the isolated suprastructures in close proximity to collagen VI-labeling, demonstrating that the two proteins are part of the same suprastructures *in vivo* (Fig. 6).

**Figure 7:**
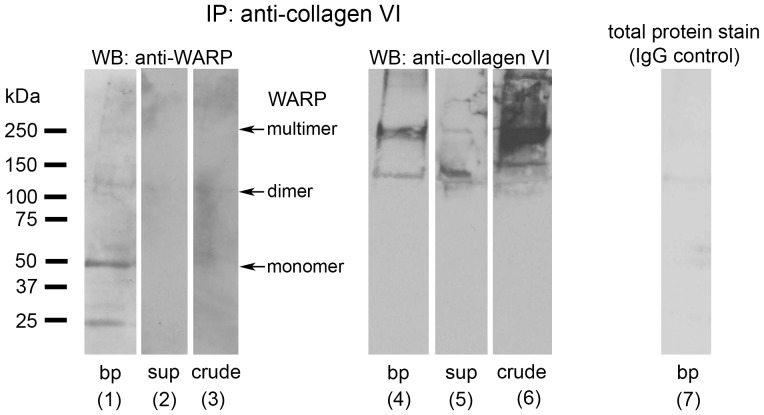
WARP co-precipitates with collagen VI. Collagen VI was isolated from cartilage extracts using magnetic beads. Following magnetic bead separation, total lysate, supernatant and pellet fractions were immunoblotted using antibodies against WARP and collagen VI. Fractions containing crude isolate (lanes 3 and 6), magnetic bead-isolated collagen VI (lanes 1 and 4), and supernatant remaining following collagen VI precipitation (lanes 2 and 5) were subjected to SDS-PAGE and immunoblotted using sheep anti-WARP antiserum (lanes 1 to 3) or anti-collagen VI antibodies (lane 4 to 6). WARP was present in collagen VI-precipitated material but not the supernatant indicating that both WARP and collagen VI are present in suprastructures. A control demonstrating that virtually no material is isolated by normal mouse serum coupled to magnetic beads is shown in lane 7 (total protein stain of blot membrane). Under reducing conditions WARP migrates as a 50 kDa monomer and the α1 and α2 chains of collagen VI co-migrate at approximately 200 kDa. Molecular weight marker (kDa) is shown on left. bp, bead pellet; sub, supernatants; crude, crude extract.

Following magnetic bead separation, total lysate, supernatant and pellet fractions were immunoblotted using antibodies against WARP and collagen VI (Fig. 7). Collagen VI was present in the total lysate and associated with the magnetic beads following separation as expected (lanes 6 and 4, respectively). Consistent with the EM data showing WARP in collagen VI suprastructures, WARP was present in the material isolated by magnetic bead separation (lane 1) but not the supernatant (lane 2). Magnetic beads labeled with mouse IgG failed to precipitate any collagen VI (lane 7). These data indicate that both WARP and collagen VI are present in suprastructures isolated with collagen VI-specific immunobeads.

In the peripheral nerves of the WARP-null mouse we found that collagen VI was markedly reduced and mislocalized [3]. Our data suggests that the high affinity interactions between WARP and collagen VI are critical for stabilizing collagen VI in peripheral nerves, since in the absence of WARP collagen VI assemblies are unstable. Using cartilage as a rich source of both macromolecules we have shown in this study that collagen VI and WARP interact *in vivo*. We demonstrated that WARP and collagen VI exist in the same location in articular cartilage using immunohistochemistry and EM methodologies. Evidence for a more direct interaction was provided by solid phase binding assays and mixing experiments using purified preparations of the two molecules. Lastly, we employed a novel immuno-bead pull-down approach to show that WARP is bound to collagen VI networks isolated from cartilage.

However, unlike in peripheral nerve, collagen VI levels appeared unaffected by the loss of WARP in developing and adult articular and vertebral cartilage as assessed by immunohistochemistry [3]. These data suggest that, although WARP and collagen VI interact in cartilage as we have shown, WARP does not have a critical role in stabilizing collagen VI in this tissue [3]. The reason for the difference between nerve and cartilage is not known but may be related to the composition of collagen VI. The purified protein used in the interaction studies contain α1, α2 and α3 chains and the antibodies used in the immunohistochemistry and EM experiments recognize primarily the same chains. Now that it is known that six collagen VI chains exist [8], [9], it is plausible that different collagen VI assemblies exist in different tissues or stages of development. For example, it is possible that the other collagen VI chains (α5 and α6) chains form different types of collagen VI isoforms or assemble in different ways with α1, α2 and α3 chains to provide different functional properties and binding partners in cartilage compared to nerve. Experiments are currently underway to explore the hypothesis that collagen VI chain composition varies between tissues.

**Figure 8:**
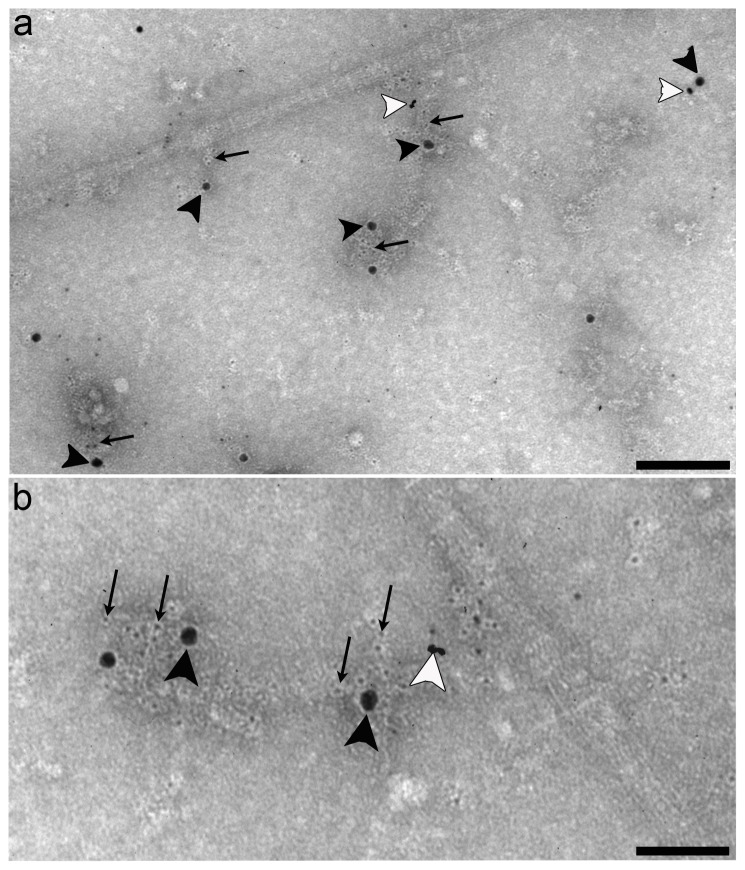
Analysis of collagen VI, WARP and perlecan in human articular cartilage. Immuno-gold EM was conducted on native supramolecular fragments isolated from human articular cartilage for WARP (18-nm gold particles; black arrowheads), perlecan (12-nm gold particles; white arrowheads) and collagen VI (6-nm gold particles, arrows). All three components are part of complexes at the suprastructural level (shown in a). The magnified image (shown in b) shows these complexes in close contact to banded collagen fibrils. Scale bars: 100 nm (panel a) and 200 nm (panel b).

We have previously reported that WARP binds to perlecan and is an integrated component of the cartilage ECM [2], [24]. Perlecan has been shown to play a major role in the mechanical and biochemical properties of the cartilage PCM [14] and, interestingly, has been shown to interact with collagen VI via its heparan sulfate domains [29]. It is possible that WARP connects the collagen VI and perlecan networks. In support of this hypothesis is a triple labeling experiment (Fig. 8) showing that collagen VI (arrows), WARP (black arrowheads) and perlecan (white arrowheads) are part of the same extrafibrillar suprastructural complexes in human articular cartilage. We suggest that by interacting with perlecan and collagen VI, WARP may function as an adaptor to bridge the pericellular perlecan network with the underlying interstitial matrix via collagen VI. We speculate that different interactions between the three molecules may also allow the generation of suprastructural organizations that vary between tissues.

## Supporting Information

Figure S1 **Collagen VI and WARP preparations.** A, Collagen VI (panel a) and WARP (panel b) samples used in binding studies. Tissue-purified collagen VI resolved under reducing conditions on a coomassie blue-stained SDS-polyacrylamide gel (4.5–15%) is shown (panel a, lane 3). Immunoblotting using rabbit polyclonal collagen VI antibody (AB 7821, Millipore) confirms the presence of collagen VI (panel a, lane 1). A coomassie-stained gel of pepsin-treated collagen VI is shown in lane 2. Silver-stained gel of recombinant WARP dimers (panel b, lane 1) and dimers plus multimers are shown (lanes 2, 3). The migration position of dimers and multimers are indicated.
(TIF)

## References

[1] Allen J, Brachvogel B, Farlie P, Fitzgerald J, Bateman J (2008) The extracellular matrix protein WARP is a novel component of a dinstinct subset of basement membranes. Matrix Biology 27: 295–305. 18314316 10.1016/j.matbio.2008.01.005

[2] Allen JM, Bateman JF, Hansen U, Wilson R, Bruckner P, et al (2006) WARP is a novel multimeric component of the chondrocyte pericellular matrix that interacts with perlecan. J Biol Chem 281: 7341–7349. 16407285 10.1074/jbc.M513746200

[3] Allen JM, Zamurs L, Brachvogel B, Schlotzer-Schrehardt U, Hansen U, et al (2009) Mice lacking the extracellular matrix protein WARP develop normally but have compromised peripheral nerve structure and function. J Biol Chem 284: 12020–12030. 19279005 10.1074/jbc.M806968200 PMC2673271

[4] Fitzgerald J, Ting ST, Bateman JF (2002) WARP is a new member of the von Willebrand factor A-domain superfamily of extracellular matrix proteins. FEBS Lett 517: 61–66. 12062410 10.1016/s0014-5793(02)02579-6

[5] Chu ML, Mann K, Deutzmann R, Pribula-Conway D, Hsu-Chen CC, et al (1987) Characterization of three constituent chains of collagen type VI by peptide sequences and cDNA clones. EurJBiochem 168: 309–317. 10.1111/j.1432-1033.1987.tb13422.x 3665927

[6] Chu ML, Pan TC, Conway D, Kuo HJ, Glanville RW, et al (1989) Sequence analysis of alpha 1(VI) and alpha 2(VI) chains of human type VI collagen reveals internal triplication of globular domains similar to the A domains of von Willebrand factor and two alpha 2(VI) chain variants that differ in the carboxy terminus. EMBO J 8: 1939–1946. 2551668 10.1002/j.1460-2075.1989.tb03598.x PMC401054

[7] Chu ML, Zhang RZ, Pan TC, Stokes D, Conway D, et al (1990) Mosaic structure of globular domains in the human type VI collagen alpha 3 chain: similarity to von Willebrand factor, fibronectin, actin, salivary proteins and aprotinin type protease inhibitors. EMBO J 9: 385–393. 1689238 10.1002/j.1460-2075.1990.tb08122.x PMC551678

[8] Gara SK, Grumati P, Urciuolo A, Bonaldo P, Kobbe B, et al (2008) Three novel collagen VI chains with high homology to the alpha3 chain. J Biol Chem 283: 10658–10670. 18276594 10.1074/jbc.M709540200

[9] Fitzgerald J, Rich C, Zhou FH, Hansen U (2008) Three novel collagen VI chains, alpha4(VI), alpha5(VI), and alpha6(VI). J Biol Chem 283: 20170–20180. 18400749 10.1074/jbc.M710139200

[10] Furthmayr H, Wiedemann H, Timpl R, Odermatt E, Engel J (1983) Electron-microscopical approach to a structural model of intima collagen. BiochemJ 211: 303–311. 6307276 10.1042/bj2110303 PMC1154360

[11] Chu ML, Conway D, Pan TC, Baldwin C, Mann K, et al (1988) Amino acid sequence of the triple-helical domain of human collagen type VI. JBiolChem 263: 18601–18606. 3198591

[12] Ishikawa H, Sugie K, Murayama K, Ito M, Minami N, et al (2002) Ullrich disease: collagen VI deficiency: EM suggests a new basis for muscular weakness. Neurology 59: 920–923. 12297580 10.1212/wnl.59.6.920

[13] Niiyama T, Higuchi I, Suehara M, Hashiguchi T, Shiraishi T, et al (2002) Electron microscopic abnormalities of skeletal muscle in patients with collagen VI deficiency in Ullrich's disease. Acta Neuropathol 104: 67–71. 12070666 10.1007/s00401-002-0522-z

[14] Wilusz RE, Defrate LE, Guilak F (2012) A biomechanical role for perlecan in the pericellular matrix of articular cartilage. Matrix Biol 10.1016/j.matbio.2012.05.002 PMC3437241 22659389

[15] Kuo H-J, Maslen CL, Keene DR, Glanville RW (1997) Type VI collagen anchors endothelial basement membranes by interacting with type IV collagen. JBiolChem 272: 26522–26529. 10.1074/jbc.272.42.26522 9334230

[16] Hanssen E, Reinboth B, Gibson MA (2003) Covalent and non-covalent interactions of betaig-h3 with collagen VI. Beta ig-h3 is covalently attached to the amino-terminal region of collagen VI in tissue microfibrils. J Biol Chem 278: 24334–24341. 12719415 10.1074/jbc.M303455200

[17] Burg MA, Tillet E, Timpl R, Stallcup WB (1996) Binding of the NG2 proteoglycan to type VI collagen and other extracellular matrix molecules. JBiolChem 271: 26110–26116. 10.1074/jbc.271.42.26110 8824254

[18] Keene DR, Ridgway CC, Iozzo RV (1998) Type VI microfilaments interact with a specific region of banded collagen fibrils in skin. J Histochem Cytochem 46: 215–220. 9446828 10.1177/002215549804600210

[19] Wiberg C, Hedbom E, Khairullina A, Lamande SR, Oldberg A, et al (2001) Biglycan and decorin bind close to the n-terminal region of the collagen VI triple helix. J Biol Chem 276: 18947–18952. 11259413 10.1074/jbc.M100625200

[20] Poole CA, Ayad S, Schofield JR (1988) Chondrons from articular cartilage: I. Immunolocalization of type VI collagen in the pericellular capsule of isolated canine tibial chondrons. JCell Sci 90: 635–643. 3075620 10.1242/jcs.90.4.635

[21] Kvist AJ, Nystrom A, Hultenby K, Sasaki T, Talts JF, et al (2008) The major basement membrane components localize to the chondrocyte pericellular matrix–a cartilage basement membrane equivalent? Matrix Biol 27: 22–33. 17825545 10.1016/j.matbio.2007.07.007

[22] Wilusz RE, Defrate LE, Guilak F (2012) Immunofluorescence-guided atomic force microscopy to measure the micromechanical properties of the pericellular matrix of porcine articular cartilage. J R Soc Interface 10.1098/rsif.2012.0314 PMC3479909 22675162

[23] Alexopoulos LG, Youn I, Bonaldo P, Guilak F (2009) Developmental and osteoarthritic changes in Col6a1-knockout mice: biomechanics of type VI collagen in the cartilage pericellular matrix. Arthritis Rheum 60: 771–779. 19248115 10.1002/art.24293 PMC2724839

[24] Melrose J, Roughley P, Knox S, Smith S, Lord M, et al (2006) The structure, location, and function of perlecan, a prominent pericellular proteoglycan of fetal, postnatal, and mature hyaline cartilages. J Biol Chem 281: 36905–36914. 16984910 10.1074/jbc.M608462200

[25] Kassner A, Hansen U, Miosge N, Reinhardt DP, Aigner T, et al (2003) Discrete integration of collagen XVI into tissue-specific collagen fibrils or beaded microfibrils. Matrix Biol 22: 131–143. 12782140 10.1016/s0945-053x(03)00008-8

[26] Spissinger T, Engel J (1995) Type VI collagen beaded microfibrils from bovine cornea depolymerize at acidic pH, and depolymerization and polymerization are not influenced by hyaluronan. Matrix Biology 14: 499–505. 7795888 10.1016/0945-053x(95)90007-1

[27] Wiberg C, Klatt AR, Wagener R, Paulsson M, Bateman JF, et al (2003) Complexes of matrilin-1 and biglycan or decorin connect collagen VI microfibrils to both collagen II and aggrecan. J Biol Chem 278: 37698–37704. 12840020 10.1074/jbc.M304638200

[28] Villone D, Fritsch A, Koch M, Bruckner-Tuderman L, Hansen U, et al (2008) Supramolecular interactions in the dermo-epidermal junction zone: anchoring fibril-collagen VII tightly binds to banded collagen fibrils. J Biol Chem 283: 24506–24513. 18599485 10.1074/jbc.M802415200 PMC3259843

[29] Tillet E, Wiedemann H, Golbik R, Pan T-C, Zhang R-Z, et al (1994) Recombinant expression and structural and binding properties of α1(VI) and α2(VI) chains of human collagen type VI. EurJBiochem 221: 177–185. 10.1111/j.1432-1033.1994.tb18727.x 8168508

