# Supplementary figures and images for: WARP Interacts with Collagen VI-Containing Microfibrils in the Pericellular Matrix of Human Chondrocytes
Source: PLoS One. 2012 Dec 26;7(12):e52793. doi: 10.1371/journal.pone.0052793 (PMC3530481; doi:10.1371/journal.pone.0052793)

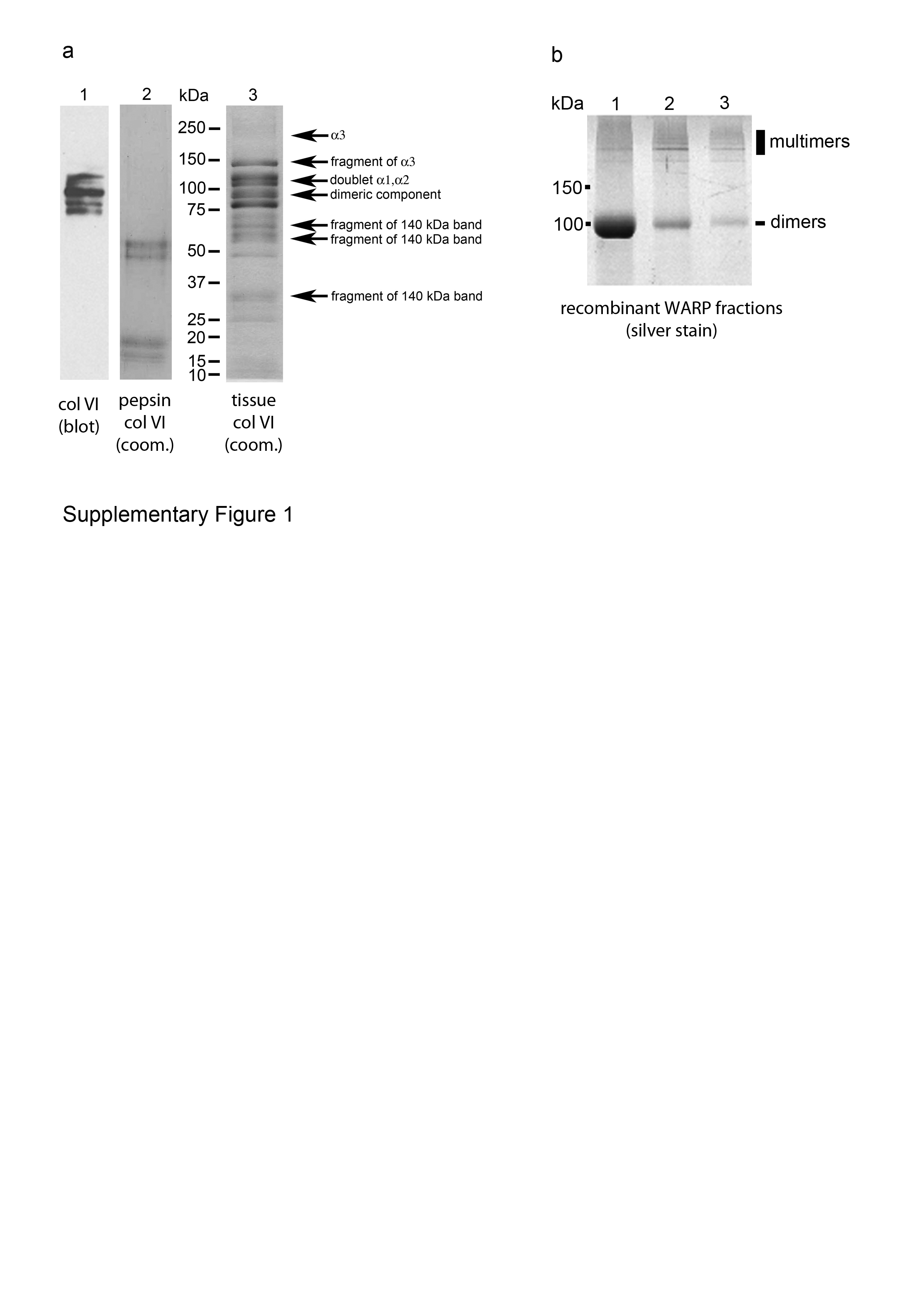

Supplement: Figure S1 — Collagen VI and WARP preparations. A, Collagen VI (panel a) and WARP (panel b) samples used in binding studies. Tissue-purified collagen VI resolved under reducing conditions on a coomassie blue-stained SDS-polyacrylamide gel (4.5–15%) is shown (panel a, lane 3). Immunoblotting using rabbit polyclonal collagen VI antibody (AB 7821, Millipore) confirms the presence of collagen VI (panel a, lane 1). A coomassie-stained gel of pepsin-treated collagen VI is shown in lane 2. Silver-stained gel of recombinant WARP dimers (panel b, lane 1) and dimers plus multimers are shown (lanes 2, 3). The migration position of dimers and multimers are indicated. (TIF) [file pone.0052793.s001.tif]
